# Prediction of bubble departing diameter in pool boiling of mixtures by ANN using modified ReLU

**DOI:** 10.1016/j.heliyon.2024.e31261

**Published:** 2024-05-22

**Authors:** Seyed Ali Alavi Fazel

**Affiliations:** Department of Chemical Engineering, Mahshahr Branch, Islamic Azad University, Mahshahr, Khuzestan, Iran

**Keywords:** Boiling, Artificial neural network, Modified activating function, Heat transfer

## Abstract

In this research, bubble departure diameter in pool boiling have been measured in aqueous amine and ethylene glycol solutions for various concentrations. The experimental data have been compared with major existing predictive correlations. It is shown that the effect and identity of the independent variables on bubble diameter proposed in the previous studies are inconsistent. The predictions of different correlations have on average a deviation of about 40% from the experimental data. This is mainly due to the complicated interactions between bubbles on the heterogeneous boiling medium, which provides a complex condition. This complexity limits any mathematical modelling of the forces acting on the developing bubbles. Particularly in liquid solutions, where mass transfer by back diffusion through micro-sub-layers adds further complexity. In this work, the classical artificial neural network, ANN, with rectified linear unit, ReLU, activating function, AF, has been modified. This modification is based on adding a numerical matrix to each layer to adjust the slope of AF for each neuron independently. The addition of this parameter, together with the adjustment of the bias matrix, makes the activation function more flexible than the classical ReLU. To find the tuning parameters, a genetic algorithm was implemented instead of the back-propagation technique. It is shown that the predictions of the trained ANN with modified ReLU AF agree within an absolute average error of 10%, which is equal to the total uncertainty of the measurements. Prediction of bubble departing diameter in boiling phenomena is a key parameter for accurate design, operation and optimisation in many industrial systems.

## Highlights

• Modelling the forces acting on bubbles in pool boiling is practically impossible.
• ANN can predict the diameter as a function of heat flux, density, viscosity, surface tension and latent heat of vaporisation.
• Modified rectified linear unit AF with bias and steepness in ANN has about 10% better prediction than classical ReLU.

## Nomenclature

α: thermal diffusivity $\frac{m^{2}}{s}$
ΔH: specific heat of vaporization $\frac{J}{\mathrm{kg}}$
qA: boiling heat flux $\frac{W}{m^{2}}$
$\mu_{l}$: liquid viscosity Pa.s
ρ: Density $\frac{\mathrm{kg}}{m^{3}}$
$\rho_{l}$: liquid density $\frac{\mathrm{kg}}{m^{3}}$
$\rho_{v}$: vapour density $\frac{\mathrm{kg}}{m^{3}}$
*σ*: surface tension $\frac{N}{m}$
*θ*: Contact angle degree
*Ar*: Archimedes number []
cpl: liquid heat capacity $\frac{J}{\mathrm{kg}.K}$
db: Bubble diameter m
*Ja*: Jacob number []
kl: liquid thermal conductivit $\frac{W}{m.K}$
ks: surface thermal conductivity $\frac{W}{m.K}$
n: number of network connections
s: distance between thermocouple location and surface m
Tb: bulk temperature K
Tt: thermocouple temperature K
Tw: surface temperature K
x: input
**AF**: Activating Function
**DEA**: diethanolamine
**MEA**: monoethanolamine
**SA**: Sensitivity Analysis
**TEG**: triethyleneglycol

## 1 Introduction

Nucleate pool boiling phenomenon is widely used in many industrial processes. The heat transfer mechanism from the surface to boiling fluid is known to be a very complicated phenomenon. Design, operation, and optimization of the boiling equipment require precise prediction of the boiling heat transfer coefficient. This is to calculate the surface temperature by a known value of heat flux or vice versa. There has been a lot of research on pool boiling over the past few decades. However, the entire mechanism of pool boiling phenomenon is still not completely understood. This is due to the complexity of three interconnected bubble-related parameters: 1) departing diameter, 2) frequency, and 3) nucleation site density. In this article, the bubble departing parameter is studied as a key parameter.

**Figure 1:**
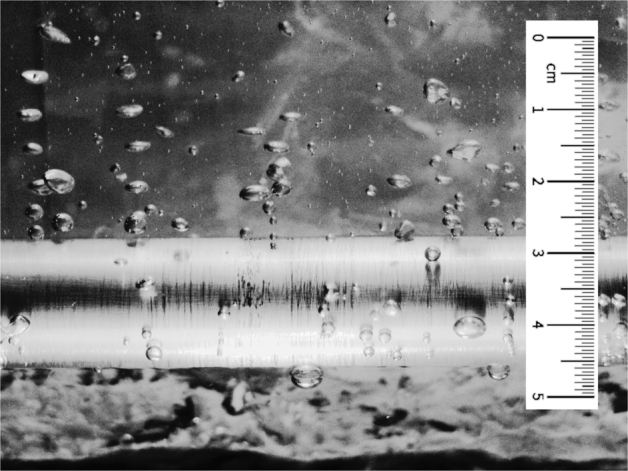
A typical boiling surface.

A typical boiling surface of pure water at a heat flux of 50 $\frac{kW}{m^{2}}$ is shown in Fig. 1. In boiling heat transfer, empirical correlations cannot predict the actual boiling heat flux under any given condition. This is due to the complicated nature of the boiling phenomenon. The complexity includes bubble nucleation, generation, expansion and also many types of interactions. This complexity practically provides many limitations to apply dynamic force balance on a growing and moving bubbles.

There are many predictive empirical models and correlations for prediction of boiling heat transferase in the literate [1]. Predictions are inconsistent even for well studied systems such as water on many boiling surfaces even ot atmospheric pressure. This is due to the complected surface microstructure. This micro-structure is usually specified by roughness. The shapes and complete topography of these irregularities are not stable during boiling process and cannot be specified. These plays a critical role in bubble diameter and frequency also nucleation site density. Consequently, boiling heat transfer coefficient is affected verse time.

Bubble diameter plays an important role in modelling and predicting the amount of heat transferred from the surface to the surrounding liquid.

In boiling condition, the heating surface can be divided into two complementary zones: 1) bubble-influenced and 2) induced forced convection zones [2]. To calculate the heat transfer flux in each zone, the bubble departing diameter at boiling should be available. The heat transfer of the affected heating zone by bubbles can be calculated by superimposing the following mechanisms 1) latent heat to the evaporating microlayer, 2) transient heat conduction 3) bubble super-heating and 4) transient heat conduction due to sliding bubbles. All these mechanisms are highly integrated in the bubble departing diameter, $d_{b}$, as a result of which accurate prediction of $d_{b}$ is significant in boiling heat transfer calculations.

## 2 Literature review

### 2.1 Bubble departing diameter

The Fritz model is one of the most important published models for both pure liquids and liquid mixtures [3]. However, it does not introduce the effect of some important parameters such as heat flux.

Stephan [4] has modified the Fritz [3] model by introducing three dimensionless Jacob, Prandtl and Archimedes numbers. The Stephan [4] correlation has better agreement with many experimental data in comparison to the Fritz [3] model, however has a high deviation for boiling electrolyte solutions [5].

Van Stralen et al. [6] proposed an empirical model by considering bubble growth mechanisms. This model includes the dimensionless Jacob number and the thermal diffusivity of the solutions.

Cole [7] included the wetting properties of liquid-solid and proposed that the effect of system pressure can be represented by the modified Jacob number.

Zeng et al. [8] assumed that the dominant forces leading to bubble detachment are unsteady growth and buoyancy forces. This model was developed based on an empirical expression for bubble growth mechanisms; however, it is only applicable when specific information on the vapour bubble growth parameters is available.

Yang et al. [9] developed a new correlation considering the analogy between nucleate boiling and forced convection heat transfer. The key parameter of this model is given graphically as a function of the Jacob number and no explicit mathematical correlation is proposed.

Jamialahmadi et al. [10] developed an empirical correlation specifically for electrolyte solutions; the bubble diameter is only correlated with the heat flux. The effect of many important parameters such as electrolyte concentration, surface tension and pressure are ignored.

Alavi Fazel et al. [5] proposed a semi-empirical correlation specifically for subcooled boiling electrolyte solutions. This correlation works well when specific vapour-liquid contact angle information is available.

Phan et al. [11] modified Fritz's [3] model to propose a new correlation for organic liquids. This equation is based on the concepts of macroscopic and microscopic contact angles. The bubble departing diameter is correlated with the contact angle and the thermophysical properties of the fluid.

Kumar et al. [12] considered the effect of wall superheat and fluid properties through the Jacob number. They proposed that the gas density and latent heat in the Jacob number are partially affected by the system pressure and express the effect of solid-liquid-gas interfacial interaction on bubble detachment.

Wang et al. [13] studied the bubble dynamics and heat transfer of pool boiling at subatmospheric pressures. They proposed that the bubble growth rate increases with decreasing pressure.

Shil et al. [14] studied the enhancement of pool boiling performance of micro/non-porous coated surfaces fabricated by a novel hybrid method. In this research, high-speed visualisation is used to perform a quantitative investigation of bubble dynamics, including active nucleation site density, emission frequency, bubble departing diameter, and their effect on pool boiling heat transfer performance.

In Table 1, the mathematical equation of four existing correlations with least mean error are presented.

**Table 1:** Mathematical equation of four existing correlations with least average error.

Author	Equation	Application
Fritz [3]	$d_{b}=0.0146\theta \sqrt{\frac{2\sigma }{g(\rho_{l}-\rho_{v})}}$	Pure liquid and mixtures
Stephan [4]	$d_{b}=0.25{\left[\;{\left(\frac{Ja}{Pr}\right)}^{0.5}\frac{100000}{Ar}\sqrt{\frac{2\sigma }{g(\rho_{l}-\rho_{v})}}\right]}^{\frac{1}{2}}$	Pure liquid and mixtures
Van Stralen et al. [6]	$d_{b}=2.63{\left[\frac{Ja^{2}\alpha_{l}^{2}}{g}\right]}^{\frac{1}{3}}{\left[1+{\left(\frac{2\pi }{Ja}\right)}^{\frac{1}{2}}\right]}^{\frac{1}{4}}$	Pure liquid and mixtures
Cole [7]	$d_{b}=0.04Ja\sqrt{\frac{\sigma }{g(\rho_{l}-\rho_{v})}}$	Pure liquid and mixtures

### 2.2 Artificial neural network, ANN

In general, ANN consists of many parallel nodes that operate and communicate with each other through connecting synapses [15]. A wide variety of ANNs have been used in a wide range of applications including pattern recognition, function approximation, optimisation, simulation, prediction, automation and many other application areas [16]. The most widely used ANNs are Multi-Layer Feed-Forward Neural Network [17], General Regression Neural Network [18], Recurrent Neural Network [19], Group Method of Data Handling Neural Network [20], Auto Regressive with an Exogenous Inputs Neural Network [21] and Radial Basis Function Neural Network [22]. A comprehensive review of ANN can be found in some other published articles [23]. In ANN, the activation function, AF, plays an important role in the training of neural networks. They provide the necessary non-linearity of the model for the ability to learn complex representations. Commonly used AFs can be divided into two main categories: 1) piecewise linear function AF and 2) locally quadratic AF. A piecewise linear function is composed of a limited number of linear segments defined over an equal number of intervals and usually of equal size. Locally Quadratic AFs are any non-linear smooth activation function with a non-zero second derivative. A summary of AFs is given in Table 2.

**Table 2:** Summary of activation functions.

Name	Equation	Range
Sigmoid	$\frac{1}{1+e^{-x}}$	(-∞, +∞ )
Hyperbolic tangent	*tanh*(*x*)	[−1,+1]
ReLU	*max*(0,1)	[0,+∞)
Leaky ReLU	*max*(0,1)+*α* min(0,1)	(-∞, +∞ )
PReLU	*max*(0,1)+*α* min(0,1)	(-∞, +∞ )
ReLU6	*min*(*max*(0,*x*),6)	[0,+6)
ELU	*max*(0,*x*)+*min*(0,*α*(*e*^*x*^ − 1))	(-*α*, +∞ )
SELU	*γ* (*max*(0,*x*)+*min*(0,*α*(*e*^*x*^ − 1)))	(-*α.γ*, +∞ )
Swish	$\frac{x}{1+e^{-x}}$	(≈−0.2784, +∞ )

**Sigmoid or Logistic**: This S-shaped function is used because the range is between (0 to 1). This is why it is mainly used for models where we need to predict probability as an output. Since the probability of something only exists between 0 and 1, sigmoid is the right choice. This function is differentiable. This means that we can find the slope of the sigmoid curve at any two points. In this research, because the genetic algorithm, GA, is implemented, differentiability is not an issue. **Tanh**: The advantage is that the negative inputs are plotted strongly negative and the zero inputs are plotted near zero in the tanh graph. This function is differentiable and monotonic, while its derivative is not. The tanh function is mainly used for classification between two classes. **ReLU**: AF is the most widely used activation function. It is used in almost all convolutional neural networks or deep learning [24]; the ReLU is half rectified (from the bottom). The function and its derivative are both monotonic. However, the problem is that any negative values immediately become zero, which reduces the ability of the model to properly fit or train from the data. This means that any negative input given to the ReLU AF will immediately go to zero in the graph, which in turn will affect the resulting graph by not fitting the negative values properly. **Leaky ReLU** or Leaky Rectified Linear Unit: is a type of activation function based on a small slope for negative values instead of a flat slope. The slope coefficient is determined prior to training. This type of activation function is popular in tasks where we may suffer from sparse slopes [25]. **PReLU** or parametric rectified linear unit: is an activation function that generalises the traditional rectified unit with a slope for negative values. Formally:

f(x)=$\left\{\begin{array}{cc} x & x\geq 0\\ \alpha x & x<0 \end{array}\right\}$ The intuition is that different layers may require different types of nonlinearity. Experimentally, it has been found that PReLUs for the first layer have more positive slopes, i.e. closer to linear behaviour [26][27][28][29]. **ReLU6**: a modification of ReLU where we limit the activation to a maximum size of six. This is to increase robustness when used with low precision calculations [30][31]. **SELU**: Scaled Exponential Linear Units, is a type of AF that induces self-normalising properties. More details of the SELU activation function are given by: [32]. **Swish**: a self-gated activation function discovered using reinforcement learning [33]. The Swish function doesn't have an upper bound. Unlike ReLU, Swish is a smooth non-monotonic activation function, and similar to ReLU, it is bounded at the bottom and unbounded at the top. It is shown by Mirco Milletari et al. that Swish AF allows more consistent performance over a wide range of the network [34].

### 2.3 Genetic algorithm, ANN

Various variants of GA's have been proposed by researchers [35]. The variants of GA are broadly classified into five main categories namely, real and binary coded, multi-objective, parallel, chaotic, and hybrid GAs.

In this work, Real-coded GAs (RGAs) have been used to train the ANN. The representation of chromosomes is closely associated with real-life problems. The main advantages of RGAs are robustness, efficiency, and accuracy. Researchers are working on RGAs to improve their performance. Most of RGAs are developed by modifying the crossover, mutation and selection operators.

## 3 Experimental apparatus

**Figure 2:**
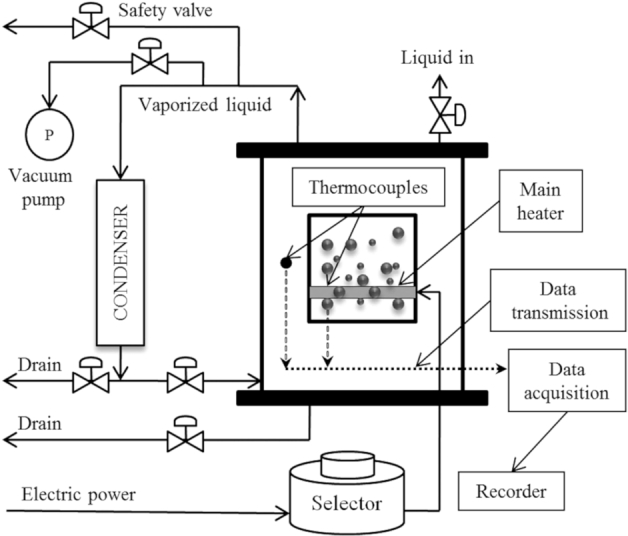
A schematic of the experimental apparatus used in this investigation.

A schematic of the experimental apparatus used in these experiments is presented at Fig. 2. The boiling vessel contains 30 litres of test solution. This volume is sufficient to provide a pool boiling condition. The vessel is thermally insulated with glass wool to minimize heat loss to the environment. An auxiliary heater was installed outside the vessel to compensate for heat loss. The temperature of system was constantly monitored and regulated to a predetermined set point. The temperature controller was manufactured by Shivaamvaj Company and connected to a K-type thermocouple. Inside the boiling tank, a rod heater with four thermocouples was placed parallel to the heating surface. After correction and calibration, the average of the four readings was taken as the surface temperature. The main heater was a 118 mm tungsten halogen linear R7s safety lamp 240 V. The input AC electrical power supply to the rod heater is adjustable via a variable electrical transformer.

**Figure 3:**
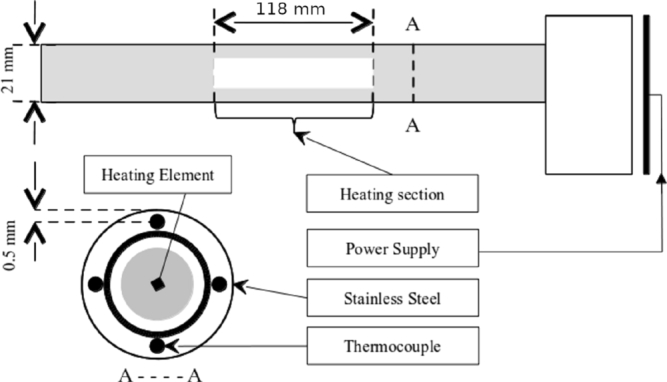
A schematic of rod heater.

The schematic of the rod heater is presented in Fig. 3. The input power of the heating rod is equal to the heat flux and is calculated by product of electrical voltage, current, and cosine of the difference between electrical voltage and current. Because the thermocouple is placed half millimetre below the heating surface, it is necessary to calculate the real surface temperature. To this end, the Fourier's conduction equation is used to calculate the temperature drop:

(1) $$T_{w}=T_{b}+(T_{t}-T_{b})\frac{s}{k_{s}}.\frac{q}{A}$$

Note that, s, the distance between thermocouple location and surface is too small (0.5 mm)and the Fourier's conduction equation is integrated in Cartesian coordination.

An average of five readings were used to determine the difference between the heating surface and the bulk temperature of each thermocouple. In this investigation, water/diethanolamine, water/triethanolamine and water/triethylene glycol solutions were used as the boiling liquid because: 1) the availability of physical properties, 2) provide a wide range of physical properties, and 3) non-toxic. The range of physical properties of the boiling fluid is shown in Table 3.

**Table 3:** Boiling liquids characteristics.

Mixture	Water	(*q*/*A*)^*max*.^	*ρ*_*l*_	*μ*_*l*_(10^−4^)	*k*_*l*_	*σ*
	%	kW/m^2^	$\frac{\mathrm{kg}}{m^{3}}$	Pa.s	$\frac{W}{m.K}$	$\frac{N}{m}$
Water/DEA	16-92	230	959-1010	2.8-6.8	0.3-0.7	0.5-0.6
Water/MEA	26-97	231	937-956	2.8-6.0	0.3-0.7	0.5-0.6
Water/TEG	16-95	264	918-979	2.8-6.0	0.2-0.6	0.5-0.6

Note that the second column is the water mole fraction. Lowest heat flux was selected at the beginning of isolated bubble regime, about 40 ${\mathrm{kW}.m}^{-2}$. A higher heat flux was selected according to the data provided in Table 3. Beyond this level, bubble diameters cannot be quantified due to interconnections. Lateral and axial interactions create a complex condition, and diameter can neither be determined nor quantified.

Concentration has been selected to provide wide range of liquid density, viscosity, thermal conductivity and surface tension as given in Table 3.

### 3.1 Uncertainty

According to the documentation of instrument manufacturer, the uncertainty of voltmeter, ammeter, millivolt meter and thermocouples used in the present study was ±1V, ±0.1A, ±0.01mV and ±0.2K respectively. The maximum expected uncertainty of four parallel thermocouples for measuring the surface temperature was estimated to (4×0.2)/4=0.2K, which means 0.2/100=0.002 or 0.2% at boiling point of pure water. Maximum expected uncertainty of boiling heat flux in terms of fraction was estimated to:

(2) $$\frac{\Delta (q/A)}{\Delta {\left(q/A\right)}_{best\;value}}=\frac{\Delta I}{I}+\frac{\Delta V}{V}=\frac{0.1}{1.1}+\frac{1}{50}=11\text{\%}$$

Note (1): assuming that maximum uncertainty in entire measurements is governed by the normal (Gaussian) distribution and that measured quantities are independent from each other, the adding in quadrature rule renders the following fraction of uncertainty:

(3) $$\frac{\Delta (q/A)}{\Delta {\left(q/A\right)}_{\mathrm{best}\;\mathrm{value}}}=\sqrt{{\left(\frac{0.1}{1.1}\right)}^{2}+{\left(\frac{1}{50}\right)}^{2}}=9\text{\%}$$

Note (2): boiling heat flux was calculated by the products of electrical current and respective voltage divided to heating area of the rod heater. The heating area was considered to be accurate enough to exclude from error analysis.

## 4 Experimental procedure

First the system was cleaned and the test solution was introduced. The temperature of the system was then adjusted to saturation point. Electrical voltage was then applied to the rod heater to provide maximum heat flux. After reaching steady state, all temperatures, electrical currents and voltages, as well as visual information, have been recorded. The electrical voltage was then decreased at various intervals and the recordings were repeated at each interval after the next steady state. Note that the decreasing order of the voltage changes was used to avoid hysteresis effects. The temperature readings from the four measurement points were within ±0.2 K of each other. It was found that the measuring points were approximately in agreement within ±2%. To measure the bubble diameters, photographs of the heating surface were taken at high speed. A Nikon D90 DSLR was used as the image recorder. For each condition, the diameters of 20 bubbles were measured and the arithmetic mean was calculated. All experiments were performed at atmospheric pressure.

## 5 Result and discussion

**Figure 4:**
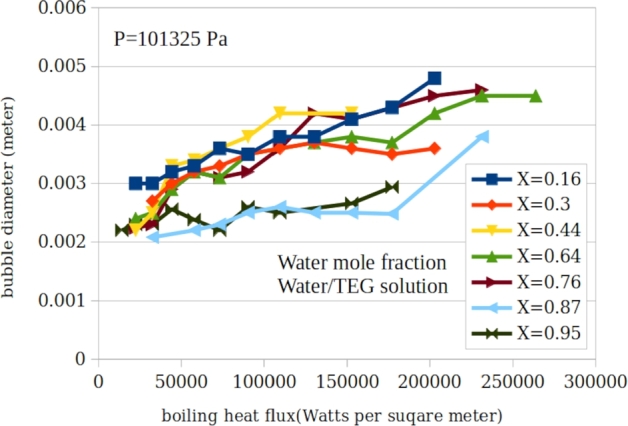
A typical presentation of measured bubble diameter as a function of heat flux for water/triethyleneglycol solution for various concentrations.

**Figure 5:**
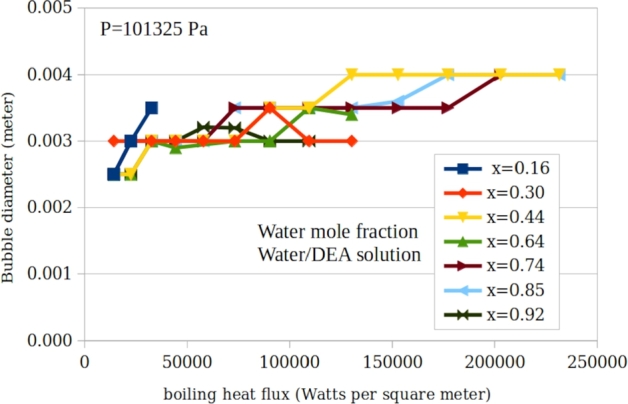
A typical presentation of measured bubble diameter as a function of heat flux for water/diethanolamine solution for various concentrations.

**Figure 6:**
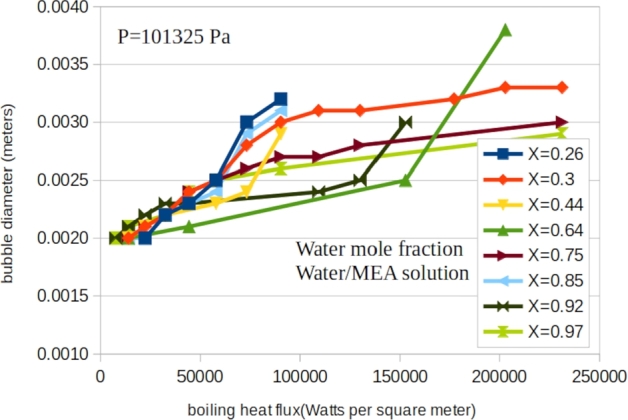
A typical presentation of measured bubble diameter as a function of heat flux for water/triethyleneglycol solution for various concentrations.

**Figure 7:**
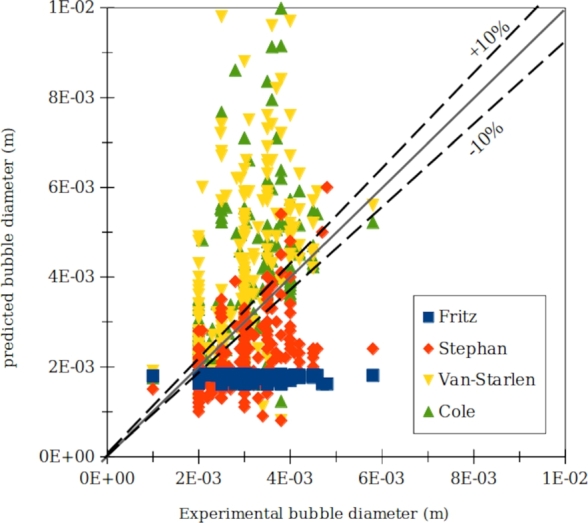
Comparison between experimental data and major existing correlations.

Figure 4, Figure 5, Figure 6 shows the measured bubble diameter as a function of heat flux for water/TEG, water/DEA and water/MEA solutions for various concentrations respectively. It is shown that the average bubble diameter increases with increasing heat flux. This is due to a higher rate of vapour generation at the micro-sublayer with a constant growth time of the attached bubbles at the surface. In Fig. 7 the experimental data are compared with the predictions of the main existing correlations. This figure includes the entire measured 163 data points for water/TEG, water/MEA and water/DEA.

Van Stralen [6] with an average absolute error, AAE, of about 59% has the largest deviation from the experimental data. The Fritz correlation [3] with an AAE of about 39% also has a high deviation from experimental data. In this correlation the bubble diameter is assumed to be independent of the heat flux. The Cole [7] and Stephan [4] correlations also have AARs of about 34% and 29% respectively. The low performance of existing correlations may be related to: 1) Complexity of surface micromorphology, 2) axial and lateral bubble interactions. None of the existing correlations take these factors into account.

Note that, in this investigation, bubbles are generated from a rod heater. Further heating surfaces may result somewhat different bubble diameters. A complete discussion about bubble generation on rod heater can be found in other articles [2].

### 5.1 Artificial neural network modelling

In this investigation, all the independent parameters for predicting of bubble diameter are: boiling heat flux, $\frac{q}{A}$, liquid bulk temperature, $T_{b}$, surface temperature, $T_{w}$, liquid and vapour densities, $\rho_{l}$ and $\rho_{v}$, liquid viscosity, $\mu_{l}$ liquid heat capacity, $c_{pl}$, liquid thermal conductivity, $k_{l}$, specific heat of vaporization, Δ*H* and surface tension, *σ*.

Based on 8 inputs, the architecture of {8,8,1}, i.e., one hidden layer with 8 neurons has been proposed and trained. The training, validating and testing AAE were 10%, 11% and 10% respectively.

**Figure 8:**
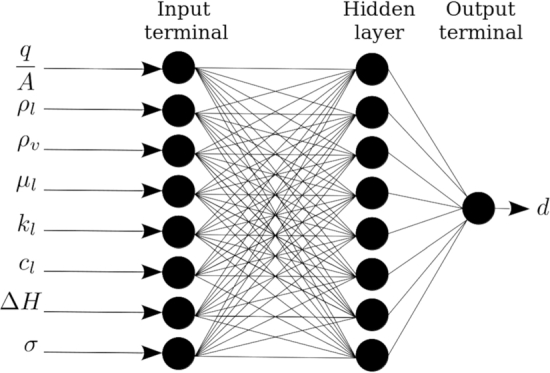
The architecture of initial artificial neural network.

**Figure 9:**
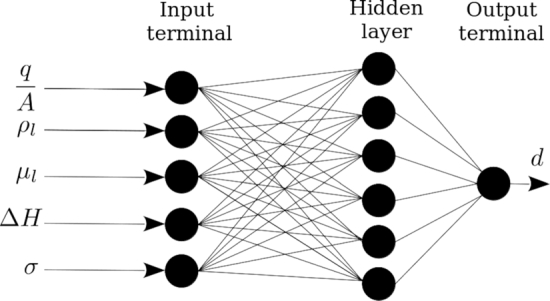
The architecture of modified artificial neural network.

**Figure 10:**
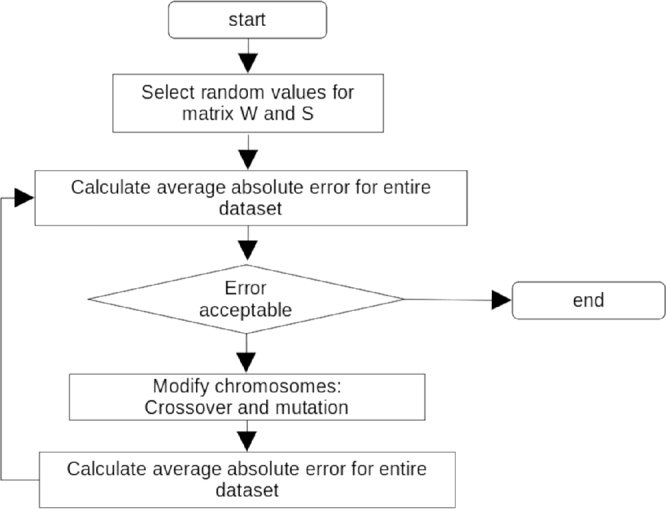
The flowchart of training ANN by genetic algorithm.

Fig. 8 shows some details of the initial artificial neural network. Note that the uncertainty of the measurements based on the Gaussian approach was about 10%. Sensitivity analysis, SA, was performed on the trained model to highlight the effective parameters and ignore the ineffective ones. Based on the SA, it was found that the liquid heat capacity $c_{pl}$, the liquid thermal conductivity $k_{l}$ and the vapour density $\rho_{v}$ have less influence on the bubble diameter than the uncertainty of the measurements. Consequently, these parameters were ignored and a modified artificial neural network with the architecture of {5,6,1}, i.e. a hidden layer with 6 neurons, was proposed and trained. The architecture is shown in Fig. 9. In Fig. 10, the flowchart of training ANN by genetic algorithm has been presented.

In this investigation, instead of classic back propagation technique for training the ANN and finding the weight matrices, genetic algorithm has been implemented. To this end, initial population including weights, slope and bias matrices have been randomly generated. The weight values have been selected by the following equation:

(4) $$w=\pm \frac{\sqrt{6}}{\sqrt{n_{i}+n_{i+1}}}$$

where $n_{i}$ is the number of incoming network connections and $n_{i+1}$ is the number of outgoing network connections from that layer. The slope and bias matrix are randomly set in range between [0.1,3] and [−5,+5] respectively. Thereafter, the total average error has been calculated by a known network. After selecting the best matrices by sorting the networks, genetic algorithm has been used including: single, two and multi-parents modification, crossover and mutation. The average error of the new generation has been recalculated until average error has been minimized. This technic would avoid falling in local minimum. The entire procedure has been written in c++ programming language.

**Figure 11:**
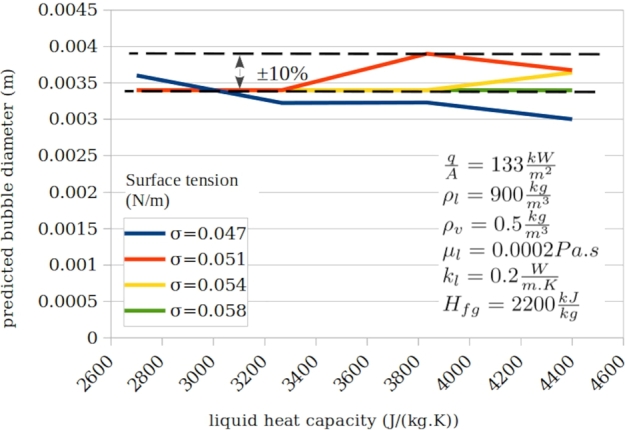
Typical impact of liquid heat capacity at various surface tensions and typical constant parameters.

**Figure 12:**
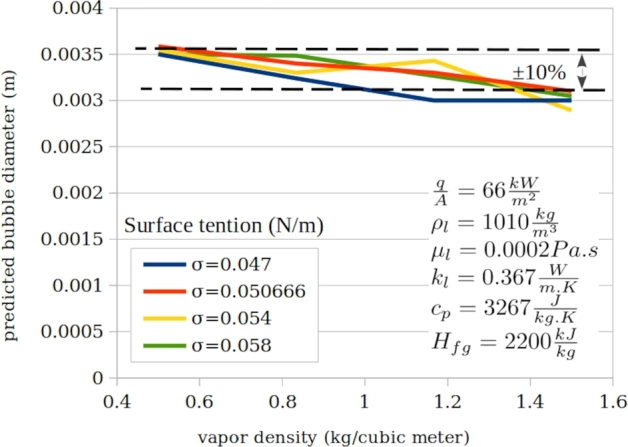
Typical impact of vapour density at various surface tensions and typical constant parameters.

**Figure 13:**
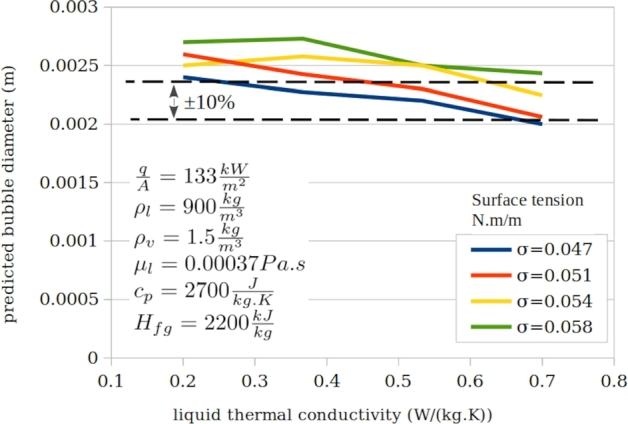
Typical impact of liquid thermal conductivity at various surface tensions and typical constant parameters.

Figure 11, Figure 12, Figure 13 presents typical impact of the three ignored parameters.

**Figure 14:**
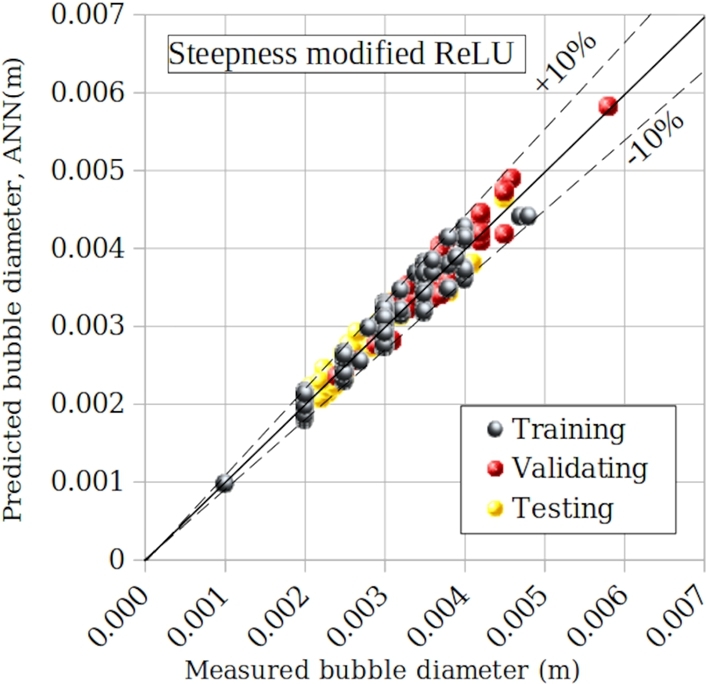
Comparison between experimental data and the new modified model.

Fig. 14 compares the experimental data with ANN model. Deviations for training, validating and testing are all within ± 10%, which is equal to the uncertainty of the new model.

**Figure 15:**
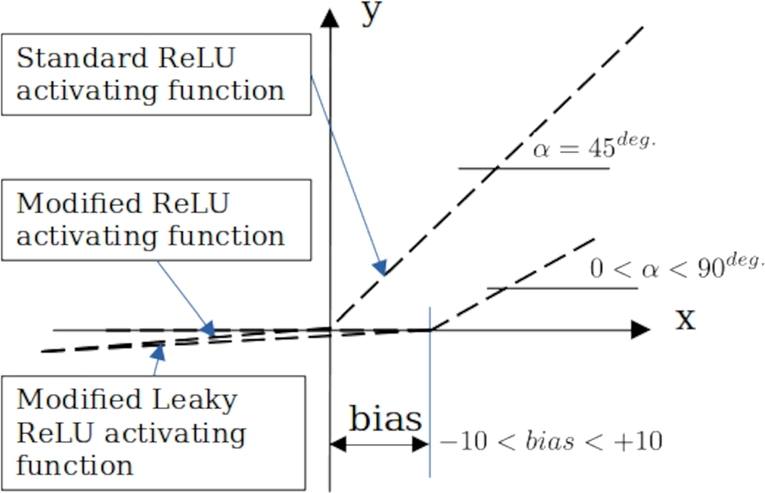
Comparison between original and modified ReLU activating function.

The new model has two advantage: 1) number of independent variables has been reduced to low as possible. 2) instead of classic ReLU activating function, which the slope in 45 degrees,

In Fig. 15, original and modified ReLU activating function are compared. Steepness adjustments provides better flexibility of the model.

The data have bee divided to 3 groups. 60% of the data for training, 20% for validating and 20% for testing. All data have been shuffled and the aforementioned groups have been chosen randomly. After each training, the data have been validated by the unseen group to select the architecture. Finally, the network has been tested by the last unseen group.

To make sure that the trained ANN has extrapolation capability, the entire procedure has been repeated six times after data shuffling.

## 6 Conclusion

In this investigation, bubble diameters were measured in pool boiling of monoethanolamine, diethanolamine and triethylene glycol at various concentrations. The experimental data were compared with the main existing correlations. It was found that the predictions are at best within ±30% AAE in accuracy. Artificial neural networks were used to model the data. Through sensitivity analysis it was found that the bubble diameter can be correlated with the boiling heat flux: boiling heat flux, $\frac{q}{A}$, liquid density, $\rho_{l}$, liquid viscosity, $\mu_{l}$, specific heat of vaporisation, Δ*H* and surface tension, *σ*. Modifying the rectifying linear unit activation function by adding a slope matrix to the neural network can improve the modelling by about 10% absolute average error.

## CRediT authorship contribution statement

**Seyed Ali Alavi Fazel:** Writing – review & editing, Writing – original draft, Visualization, Validation, Supervision, Software, Resources, Project administration, Methodology, Investigation, Funding acquisition, Formal analysis, Data curation, Conceptualization.

## Declaration of Competing Interest

The authors declare that they have no known competing financial interests or personal relationships that could have appeared to influence the work reported in this paper.
